# Hidradenitis Suppurativa Mimicking Tuberculosis Scrofuloderma: The Role of Dermatologic Ultrasound as a Diagnostic Tool

**DOI:** 10.7759/cureus.83659

**Published:** 2025-05-07

**Authors:** Julia A Maya, John V Veasey, Rute F Lellis, Priscilla T Foster, Elisete I Crocco

**Affiliations:** 1 Dermatology, Irmandade Santa Casa de Misericórdia de São Paulo, São Paulo, BRA; 2 Pathology, Irmandade Santa Casa de Misericórdia de São Paulo, São Paulo, BRA; 3 Radiology, Irmandade Santa Casa de Misericórdia de São Paulo, São Paulo, BRA

**Keywords:** dermatological ultrasound, hidradenitis suppurativa, medical dermatology, painful skin lesions, scrofuloderma

## Abstract

Hidradenitis suppurativa (HS) is a chronic inflammatory disease of the apocrine glands, typically presenting with cycles of flare-ups and periods of improvement. It predominantly affects women and is commonly found in areas such as the axillae, inguinal, and inframammary regions. We present a case of a 25-year-old male from Brazil who presented with recurrent cervical lesions over two years, initially suspected to be skin tuberculosis (scrofuloderma). Diagnostic workup included polymerase chain reaction (PCR), tuberculin skin test (TST), chest X-ray, and histological analysis, all of which ruled out infectious causes. High-frequency dermatological ultrasound revealed tortuous fistulous tracts with hyperechoic fragments in the dermis and subcutaneous tissue, suggesting HS. A subsequent biopsy confirmed the diagnosis of HS, with histopathological findings of perifollicular lymphocytic infiltration. The patient’s clinical progression and the development of a new lesion in the right axilla further supported the diagnosis. This case highlights the importance of ultrasound as an adjunctive tool in diagnosing atypical HS presentations and differentiating it from other conditions such as scrofuloderma. It also emphasizes the necessity of considering non-intertriginous areas when diagnosing HS and the need for a comprehensive diagnostic approach, including imaging and biopsy, to exclude other diseases.

## Introduction

Hidradenitis suppurativa (HS) is a chronic inflammatory disease of the apocrine glands, characterized by recurrent episodes of improvement and exacerbation [1-2]. Lesions are typically painful, with the most commonly affected areas being the axillary, inguinal, and inframammary regions [1,3]. Clinical manifestations may include deep-seated nodules, abscesses, sinus tracts (skin tunnels), and fibrotic scarring [4-5].

The disease is more prevalent in women, with onset most commonly occurring between puberty and the age of 40. Diagnosis is primarily clinical, based on the characteristic presentation, involvement of typical anatomical locations, and recurrence of lesions [6]. In cases involving atypical lesion sites or initial presentations, dermatologic ultrasound can play a key role in supporting the diagnosis [5-6].

We present a case of HS in which ultrasonographic evaluation, in conjunction with other complementary examinations, helped confirm the diagnosis of HS in an atypical presentation site.

## Case presentation

A 25-year-old male patient, originally from Brazil, reported recurrent infections in the cervical region for the past two years, necessitating the use of multiple antibiotics and drainage of secretions. The most recent recurrence occurred about one month before admission, with local progression of lesions in the cervical region. The patient did not present with fever or weight loss, denied any ongoing acute illness or trauma, and reported no current medications, prior incarceration, or contact with individuals with tuberculosis.

**Figure 1:**
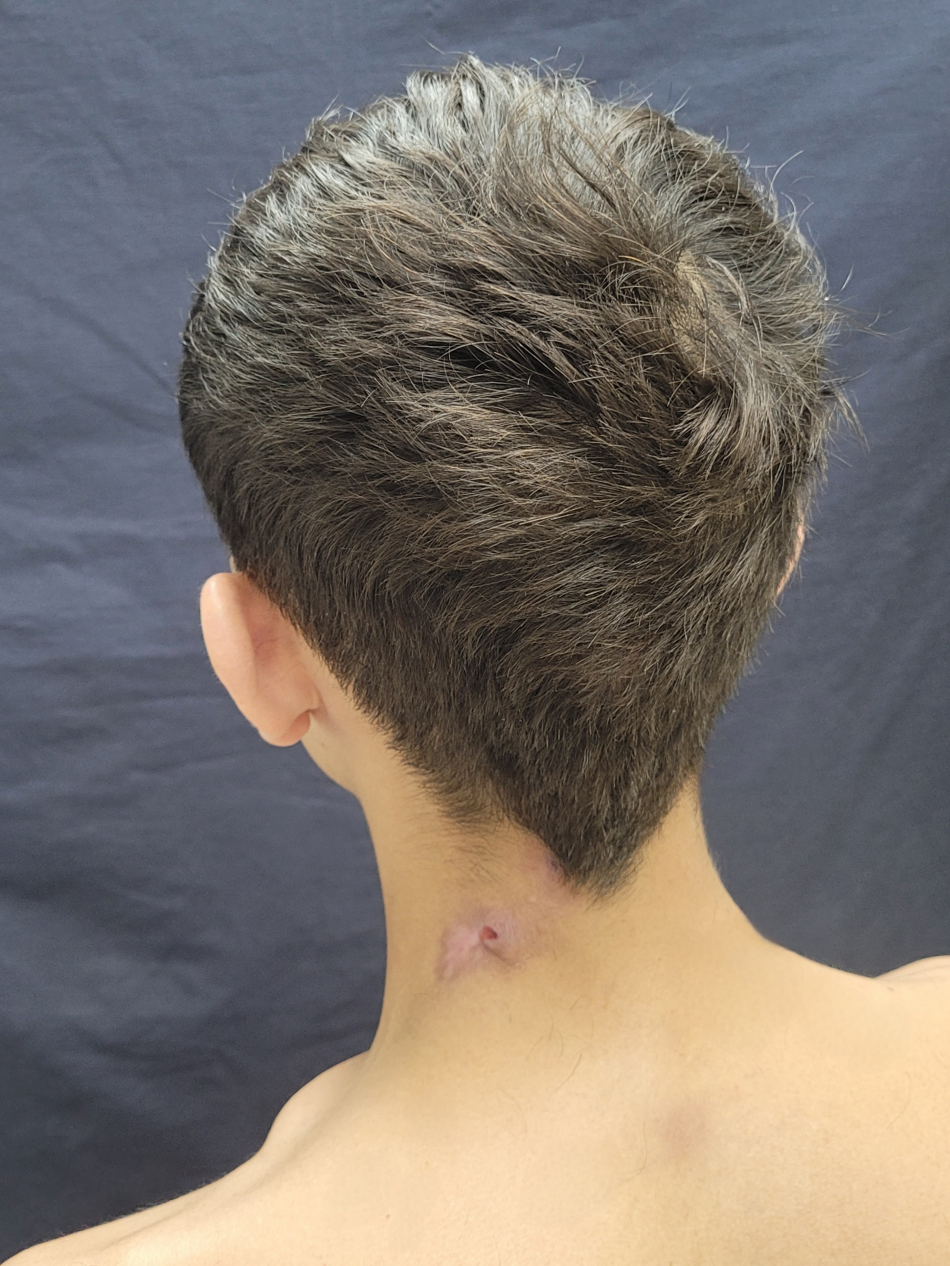
Atypical presentation of hidradenitis suppurativa. The patient upon admission to the service presented with two nodules in the posterior cervical region.

Upon clinical examination, the patient had two nodules in the cervical region, interconnected by a fistulous tract, with seropurulent content emerging from drainage orifices upon manual expression (Figure 1). Due to the patient's country of origin and the history of multiple antibiotic treatments without resolution, the diagnostic hypothesis of skin tuberculosis (scrofuloderma) was considered. 

Tests were requested, including polymerase chain reaction (PCR) with GeneXpert, tuberculin skin test (TST), chest X-ray, general laboratory tests, and serologies for hepatitis and HIV, to investigate tuberculosis and potential related comorbidities. A biopsy was also performed, with culture for fungi and bacteria from the seropurulent material collected from the patient’s clinical lesion. GeneXpert and TST presented negative results, and cultures showed no growth. The chest X-ray had no alterations, general laboratory tests were unremarkable, and serologies for hepatitis and HIV were non-reactive, ruling out infectious diseases.

**Figure 2:**
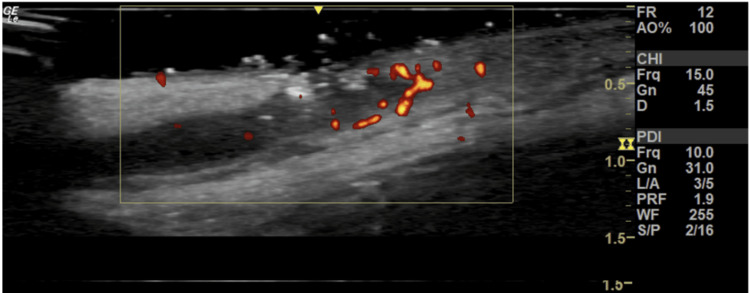
Hidradenitis suppurative ultrasound image showing hypoechoic fistulous tract with hyperechoic fragments, with increased vascularity observed on Doppler.

A high-frequency dermatological ultrasound was performed to determine the extent of the lesion and assist in the diagnostic definition. The ultrasound revealed tortuous fistulous tracts in the dermis and subcutaneous, with hyperechoic fragments within (some linear, possibly corresponding to hair shafts and keratin), with increased vascularity observed on Doppler study connecting the mid-occipital region with the left posterior cervical region and the upper dorsal region of the midline, suggesting a case of HS (Figure 2).

**Figure 3:**
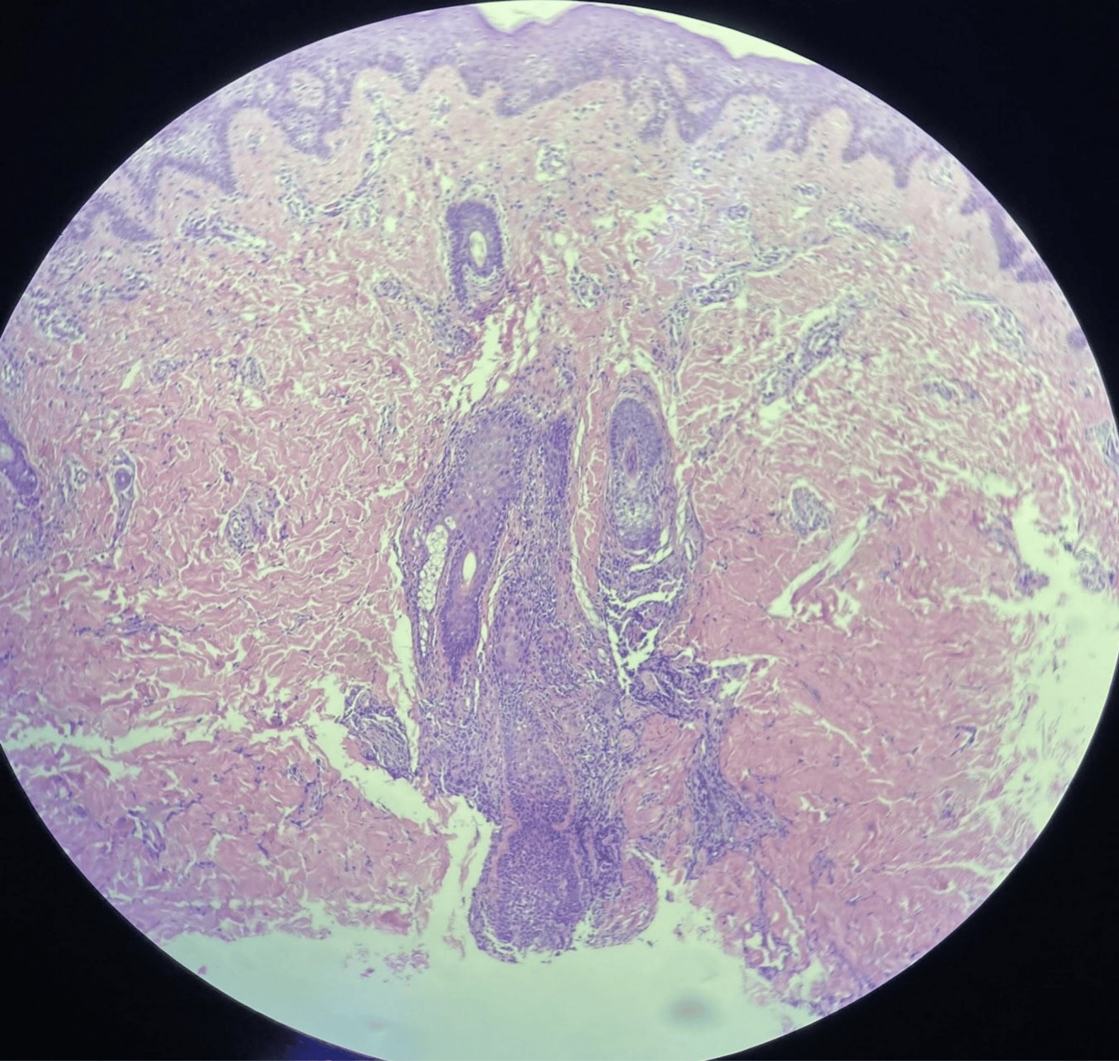
Hidradenitis suppurativa histopathological examination showing perifollicular infiltrate of lymphocytes with some suppurate foci.

In order to confirm the diagnosis, a 3-mm punch biopsy was performed. The histopathological examination revealed a perifollicular lymphocytic infiltrate with some suppurative foci. No pathogens were identified in the sample (Figure 3). Although specific features of HS, such as sinus tracts and follicular plugs, were not observed, this absence is likely due to sampling limitations inherent to the technique used. Nevertheless, the nonspecific findings are compatible with and support the clinical diagnosis of HS.

**Figure 4:**
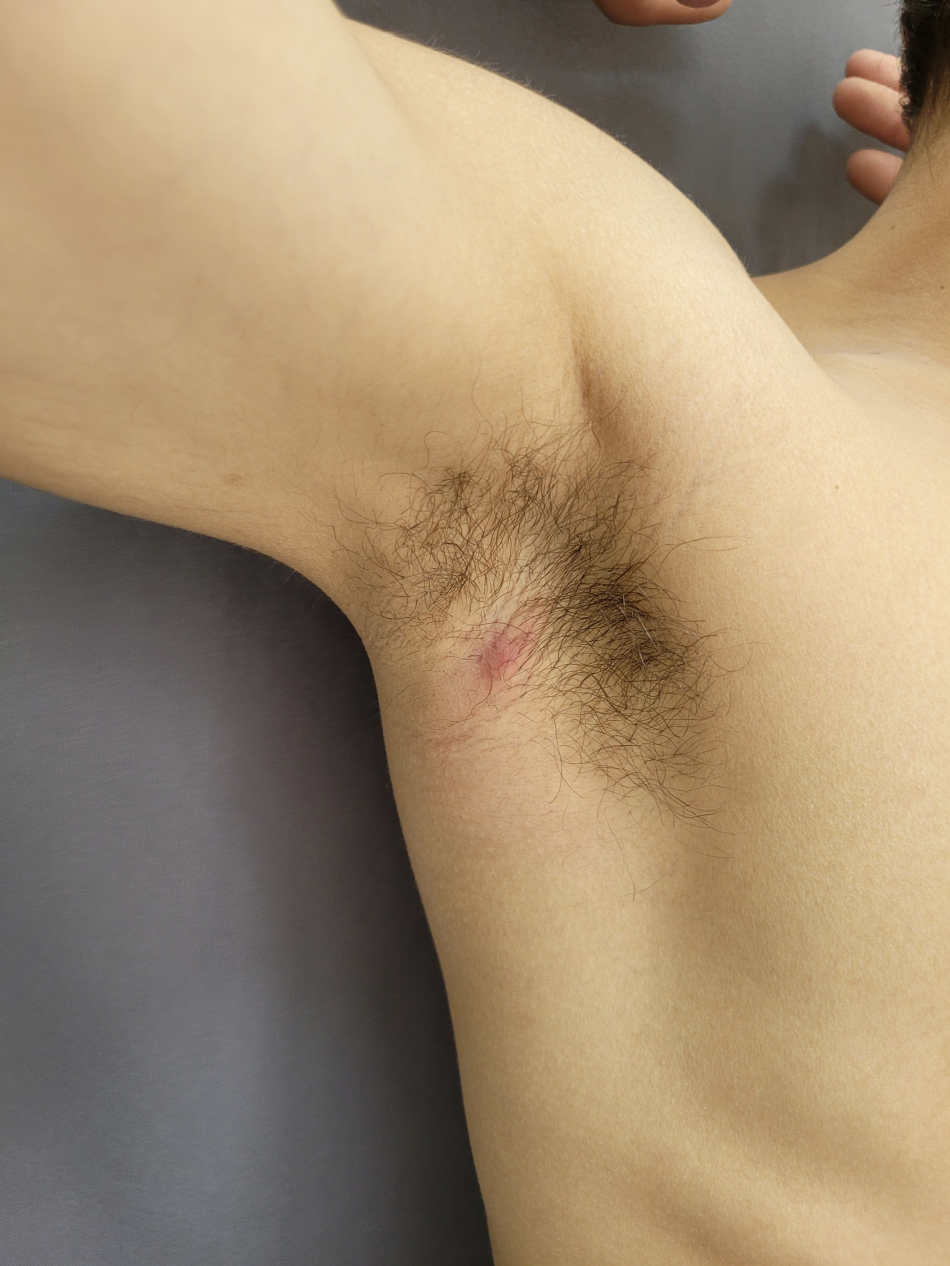
Typical presentation of hidradenitis suppurativa at the four-month follow-up.

On follow-up, four months after the initial assessment, the patient returned with a new lesion in the right axilla, aiding in the diagnostic confirmation of the disease presentation in a typical area (Figure 4).

## Discussion

Atypical forms of HS are described in non-intertriginous areas where hair follicles are present [3-6]. The disease is characterized by nodules that can evolve into abscesses and the formation of fistulas. It is usually exacerbated by smoking and obesity and is correlated with a higher tendency to develop metabolic syndrome [7-8]. Despite the patient presenting clinical lesions suggestive of the disease, these were located in an atypical area, and he did not exhibit additional clinical stigmata associated with the condition.

Scrofuloderma is a subtype of cutaneous tuberculosis that progresses with the formation of nodules and fistulous tracts containing purulent material. The most common sites of involvement are the head and neck. It can evolve insidiously and may not respond to traditional antibiotic treatments [5,7]. Pulmonary involvement and elevated PPD are usually present. Clinical lesions manifested by scrofuloderma can mimic various skin diseases, making it necessary to rule out the diagnosis in areas with a high prevalence of this disease [9-10].

To assist with the differential diagnosis between scrofuloderma and HS, key clinical findings are summarized in Table 1 [11-12].

**Table 1:** Clinical findings differentiating between scrofuloderma and hidradenitis suppurativa Adapted from Braun-Falco's Dermatology, 2022 [11-12]

Feature	Scrofuloderma	Hidradenitis Suppurativa
Affected population	Mostly children and older adults	More common in females; onset at or after puberty
Associated condition	Tuberculosis	Not associated with tuberculosis; often linked to metabolic syndrome or hormonal factors
Onset and early lesions	Visible lymph node swelling over weeks to months	Inflammatory nodules and sterile abscesses
Progression	Abscesses, induration, fistulas with skin breakdown	Sinus tracts, hypertrophic scars, malodorous drainage
Tuberculin skin test	Positive	Negative
Typical locations	Lymph node regions: cervical, axillary, thoracic, inguinal	Intertriginous areas: armpits, neck, scalp, submammary, groin, perineum, genitals
Recurrence/chronicity	New lesions can occur at previously healed sites	Chronic with recurring lesions
Scarring	Sclerosis, scars, keloids	Hypertrophic scarring

Thus, cutaneous tuberculosis in the form of scrofuloderma was initially the most relevant clinical hypothesis, as the patient was from a high-risk area for the disease and presented with clinical lesions characteristic of the disease in the most common sites, despite denying known contact with carriers.

Skin biopsy is usually not required to confirm the diagnosis of HS; however, in cases in which the diagnosis is uncertain, a biopsy can be used to exclude other causes such as pyoderma gangrenosum, lymphomas, and squamous cell carcinoma. Findings related to HS depend on the type of alteration in the biopsied area, which may show a cyst, nodule, or abscess in an inflamed sinus tract or a scar resembling a cord [6,8].

The use of high-frequency ultrasound in clinical research shows the occurrence of multiple retained hair follicles, confirming the role of hair follicles as foreign bodies that contribute to the perpetuation of inflammation in areas with HS. On ultrasound imaging, vellus hairs appear as bilaminar hyperechoic structures located between cysts or fistulas, oriented parallel to the skin surface, unlike unaffected hair follicles, which are oriented perpendicularly. In the patient, the ultrasound evaluation found a fistulous tract connecting the nodules interspersed with hair follicles [13-14].

## Conclusions

In summary, this case underscores the diagnostic challenges posed by skin diseases with similar clinical presentations, such as HS and scrofuloderma, particularly when they occur in atypical locations. Although the initial clinical findings suggested a potential diagnosis of cutaneous tuberculosis, diagnostic tools, including high-frequency dermatological ultrasound and biopsy, ultimately confirmed HS in an unusual site. The clinical progression, coupled with the appearance of new lesions in more typical areas, highlights the importance of a comprehensive diagnostic approach, including imaging and biopsy, to ensure accurate diagnosis and guide appropriate treatment.
